# The Epidemiology of Respiratory Syncytial Virus: New Trends and Future Perspectives

**DOI:** 10.3390/v16071100

**Published:** 2024-07-08

**Authors:** Irene Raffaldi, Emanuele Castagno

**Affiliations:** Department of Pediatric Emergency, Regina Margherita Children’s Hospital, 10126 Turin, Italy; iraffaldi@cittadellasalute.to.it

RSV (respiratory syncytial virus) is a major cause of acute lower respiratory tract infection (LRTI) worldwide. Immunocompromised adults and the elderly are susceptible to severe infection and high mortality. Among children, RSV is the most frequent cause of LRTI, particularly in infants and toddlers younger than 2 years old, and one half of this group will have been infected twice by this age [1,2].

Human RSV is an enveloped, spherical RNA virus belonging to the genus *Orthopneumovirus* in the family *Pneumoviridae* [1]; when infection develops, RSV forms large cells, known as syncytia. The structure of the virus consists of three membrane proteins: a small hydrophobic (SH) protein, an attachment glycoprotein (G), and a fusion (F) protein. Based on the protein G sequence, subtypes A and B can be identified. Both subtypes circulate simultaneously during an epidemic season, but usually one of the two predominates each year. The replication cycle of this virus is error-prone, allowing for a rapid generation of mutations, thus resulting in changes in the virulence of RSV and difficulties in creating antiviral agents or vaccines [3].

Its physiopathology consists of the disruption of the alveolar epithelium; this creates submucosal oedema and cilia loss on the apical surface of infected epithelial cells, together with an accumulation of mucus and cellular debris, inducing airway plugging and neutrophil infiltration in the airways. The severity of its clinical manifestations directly correlates with the virus titer and the exacerbated proinflammatory cytokine/chemokine response skewed toward a T helper type 2 immune response. However, the relative contributions of these factors to the determination of clinical features remain unknown [4].

Most children infected with RSV typically exhibit mild respiratory symptoms. The severe manifestations of RSV include pneumonia and bronchiolitis; the latter is usually self-limiting but accounts for a significant number of hospitalizations and admissions to pediatric intensive care units (PICUs), even in previously healthy, full-term newborns and infants [3].

Before the COVID-19 pandemic, RSV was typically described as a seasonal virus, characterized by a predictable epidemiological pattern, depending on the geographic area and climate [3]. In the Northern Hemisphere, the virus usually spread between November and March, with peak incidences in January/February, whereas in the Southern Hemisphere, RSV season typically occurred between June and September [3].

During the COVID-19 pandemic, drastic interventions were adopted on a global scale, and these included social distancing, stay-at-home orders, wearing face masks, and the promotion of hand washing. Such non-pharmaceutical interventions limited the circulation of SARS-CoV-2, together with other viruses transmitted through aerosol, droplets, and direct contact, such as RSV. In 2020, many countries saw an absence of RSV circulation during the “traditional” epidemiological season. For example, in Western Australia, a decrease of 98% in the detection of RSV infection was reported, compared to previous winter seasons between 2012 and 2019 [5]. Similarly, other authors reported a marked reduction in RSV spread in Europe and in other regions of the Northern Hemisphere, with a complete absence of cases noted in some countries [6,7,8,9,10].

After the initial reduction in RSV circulation, many authors recorded an out-of-season surge of RSV as the distancing measures adopted during the COVID-19 pandemic loosened. For example, in the Southern Hemisphere, a sharp rebound was observed in South Africa and New Zealand in late 2020 and early 2021, respectively [2]. Additionally, in the Northern Hemisphere, a similar rebound was also reported in Japan [11,12,13]; in the USA, where the total number of RSV infections had remained lower than expected until early 2021, a significant increase in reported infections was shown in statistics for the summer of 2021, largely before the traditional RSV outbreak period [11,12]. As regards Europe, the number of visits to the emergency department for acute bronchitis or bronchiolitis in England diverged from the seasonal trend, beginning in week 22 of 2021 and continuing onwards. In this survey, during the summer of 2021, the authors observed 9789 (84.9% [84.5 to 85.4]) more admissions than expected [11,14]. In addition to this, an Italian multicenter study reported a significantly higher admission rate for RSV infections in 2021 compared to 2020 and 2019 [3].

Epidemiological surveillance suggested that this new trend in the epidemic wave was not only present in children. During the second part of 2021, an inter-seasonal rise in RSV cases was observed even in younger adults, while an upsurge in older adults was observed later on [15].

Concerning pediatric patients in particular, some evidence for a correlation between affected patients of different ages and a higher severity of RSV-related clinical pictures arose after the first wave of the COVID-19 pandemic. On the one hand, Pruccoli et al. reported a shift towards older children presenting with symptomatic RSV infections [3]. Such an age shift was also confirmed by another report in Iceland; the median age of RSV-positive cases was 16 months during the 2020–2021 season, compared to 5.7 months across the five previous seasons [8,16].

On the other hand, Cai et al. recorded higher levels of severity in RSV-related disease and reported a percentage of 8.5% of RSV patients admitted to the PICU in 2022–2023, compared to 6.8% in pre-COVID-19 seasons. The authors also described an increased need for respiratory support than was previously reported (6.1% vs. 3.8%, respectively) [17]. However, such an observation has not been noticed by other authors, and this requires further confirmation in future epidemic seasons [3,18,19].

The COVID-19 pandemic scenario has offered a unique opportunity for researchers to learn more about the transmission of RSV and other respiratory viruses, as well as design future preventive strategies regarding RSV spread in both children and adults. Monitoring RSV seasonality is essential for pediatric health care in order to plan the appropriate timing of preventive strategies. The introduction of prophylactic measures, such as nirsevimab administration to newborns and infants, as well as the immunization of pregnant women, could be greatly influential in managing the next epidemic seasons from both clinical and organizational perspectives.
